# Association between water, sanitation and hygiene (WASH) and child undernutrition in Ethiopia: a hierarchical approach

**DOI:** 10.1186/s12889-022-14309-z

**Published:** 2022-10-19

**Authors:** Biniyam Sahiledengle, Pammla Petrucka, Abera Kumie, Lillian Mwanri, Girma Beressa, Daniel Atlaw, Yohannes Tekalegn, Demisu Zenbaba, Fikreab Desta, Kingsley Emwinyore Agho

**Affiliations:** 1 Department of Public Health, Madda Walabu University Goba Referral Hospital, Bale-Goba, Ethiopia; 2 College of Nursing, University of Saskatchewan, Saskatoon, Canada; 3 School of Public Health, College of Health Science, Addis Ababa University, Ababa, Ethiopia; 4 Torrens University Australia, Adelaide Campus, 5000 Adelaide, SA Australia; 5 Department of Human Anatomy, Madda Walabu University Goba Referral Hospital, Bale-Goba, Ethiopia; 6 School of Health Sciences, Western Sydney University, Locked Bag 1797, 2751 Penrith, NSW Australia

**Keywords:** EDHS data, Stunting, Wasting, Under-five children, WASH, Hierarchical models, Ethiopia

## Abstract

**Background:**

Undernutrition is a significant public health challenge and one of the leading causes of child mortality in a wide range of developing countries, including Ethiopia. Poor access to water, sanitation, and hygiene (WASH) facilities commonly contributes to child growth failure. There is a paucity of information on the interrelationship between WASH and child undernutrition (stunting and wasting). This study aimed to assess the association between WASH and undernutrition among under-five-year-old children in Ethiopia.

**Methods:**

A secondary data analysis was undertaken based on the Ethiopian Demographic and Health Surveys (EDHS) conducted from 2000 to 2016. A total of 33,763 recent live births extracted from the EDHS reports were included in the current analysis. Multilevel logistic regression models were used to investigate the association between WASH and child undernutrition. Relevant factors from EDHS data were identified after extensive literature review.

**Results:**

The overall prevalences of stunting and wasting were 47.29% [95% CI: (46.75, 47.82%)] and 10.98% [95% CI: (10.65, 11.32%)], respectively. Children from households having unimproved toilet facilities [AOR: 1.20, 95% CI: (1.05,1.39)], practicing open defecation [AOR: 1.29, 95% CI: (1.11,1.51)], and living in households with dirt floors [AOR: 1.32, 95% CI: (1.12,1.57)] were associated with higher odds of being stunted. Children from households having unimproved drinking water sources were significantly less likely to be wasted [AOR: 0.85, 95% CI: (0.76,0.95)] and stunted [AOR: 0.91, 95% CI: (0.83, 0.99)]. We found no statistical differences between improved sanitation, safe disposal of a child’s stool, or improved household flooring and child wasting.

**Conclusion:**

The present study confirms that the quality of access to sanitation and housing conditions affects child linear growth indicators. Besides, household sources of drinking water did not predict the occurrence of either wasting or stunting. Further longitudinal and interventional studies are needed to determine whether individual and joint access to WASH facilities was strongly associated with child stunting and wasting.

**Supplementary Information:**

The online version contains supplementary material available at 10.1186/s12889-022-14309-z.

## Introduction

Undernutrition, which includes stunting (low height-for-age), wasting (low weight-for-height), and underweight (low weight-for-age), is one of the major public health problems and makes children under-five years of age (under-fives) in particular, more vulnerable to disease and death. Stunting results from chronic or recurrent undernutrition, whereas wasting usually indicates recent and severe weight loss because a person has not had sufficient food intake and/or has had an infectious disease, such as diarrhea, resulting in rapid weight loss [1].

Early childhood linear growth is a strong indicator of healthy growth and is linked to child development in several domains, including cognitive, language, and sensory-motor capacities [2]. Globally, in 2020, 149 million under-fives were estimated to be stunted (too short for their age), and 45 million were estimated to be wasted (too thin for height). Undernutrition was reported to be responsible for approximately 45% of deaths among under-fives in low- and middle-income countries (LMICs), with Sub-Saharan Africa (SSA) bearing the greatest burden [1, 3, 4].

Undernutrition remains pervasive, with stunting, wasting, and underweight highly prevalent in SSA [3, 4]. Previous studies have shown this region to have the highest prevalence of stunting at 32% [5], underweight at 16.3%, and wasting at 7.1% [4]. A closer examination showed that the prevalence of malnutrition was highest in Eastern African countries, including Ethiopia [4, 6].

According to the 2019 Ethiopian Mini Demography and Health Surveys (EDHS) report, 37% of under-fives were stunted, 21% were underweight, and 7% were wasted [7]. In Ethiopia, several primary studies have also revealed that the prevalence of stunting and wasting in children ranges from 49.2 to 58.1% [8–12] and 13–17% [10–12], respectively. A systematic review conducted in Ethiopia showed that the overall pooled prevalence estimates of stunting, underweight, and wasting were 34.42%, 33.0%, and 15.0%, respectively [6].

In Ethiopia, several studies have identified the predictors of childhood undernutrition, revealing factors associated with stunting as the age of the child [8, 9], households that did not treat drinking water at the point of use [8], access to combined improved WASH facilities [10], lack of improved sanitation facilities [11], maternal body mass index (BMI) [8, 11, 12], lack of maternal education [13, 14], poor wealth status [13–15], house main floor material [13], lack of exclusive breastfeeding among infant under 6 months of age [12], no intake of meat by a child [12], birth size [13, 14], birth order [9], short birth interval [11], and a child having repeated diarrheal episodes [12]. Similarly, different studies elicited the predictors of wasting in children ,including: being born smaller than average size [10], sex of the child [8, 16], cough [8], fever [17], maternal body mass index [10], maternal education [8, 18], maternal occupation [8], diarrheal morbidity [16, 18, 19], and initiating complementary food before 6 months of age [18]. The evidence further indicates that children with poor access to proper WASH are likely to experience impaired child growth [20]. However, in SSA, studies on the effects of WASH on child growth are limited [21–24]. In Ethiopia, scientific evidence explicitly focusing on the relationship between WASH and childhood malnutrition is scarce [10, 25, 26].

Previous studies using EDHS datasets were surveyed specifically and focused on socioeconomic inequality [27], stunting [13, 14, 17], trends in child growth failure [28], investigating spatial variations [11], and focusing on concurrent nutritional deficiencies [9]. Besides, there is no quantitative pooled data evidence on the association between WASH and childhood undernutrition [10, 11].

Because malnutrition, especially undernutrition, remains endemic in Ethiopia, further evidence is needed to identify the links between WASH and both acute and chronic malnutrition in order to inform future directions for research in this area. This study aimed to assess the association between WASH and undernutrition (wasting and stunting) among under-fives in Ethiopia. Findings from this study will potentially inform and enable policymakers and public health researchers to target vulnerable children in the population for future interventions.

## Methods

### Study setting

Ethiopia is Africa’s second-most populated country, after Nigeria, with a population of over a hundred million people. Ethiopia, with a federal system of government has 10 regions (i.e., Afar, Amhara, Benishangul-Gumuz, Gambella, Harari, Oromia, Somali, Sidama, Southern Nations and Nationalities and People (SNNP), and Tigray) and two chartered cities (i.e., Addis Ababa and Dire Dawa). Ethiopia shares borders with Eritrea in the north, Kenya and Somalia in the south, South Sudan and North Sudan in the west, and Djibouti and Somalia in the East [29].

### Data source

The datasets from the four rounds of the Ethiopian Demography and Health Surveys (EDHS) conducted from 2000 to 2016 were used in this study [29–32]. The EDHS is a nationally representative survey collected every five years, providing population and health indicators at the regional and national levels. The EDHS used a multistage cluster sampling technique, whereby data are hierarchical (i.e., children and mothers were nested within households, and households were nested within clusters). For this reason, we employed a multilevel logistic regression model, which has many advantages over the classical logistic regression model and is appropriate for analysing factors from different levels. A detailed description of analysis is presented in the data analysis section. The datasets of each survey were obtained from the following EDHS data repository https://dhsprogram.com.

### Sampling and data collection

In brief, the 2000 and 2005 data were collected based on the 1994 population and housing census frame, while the 2011 and 2016 data were collected based on the 2007 population and housing census frame [29–32]. EDHS data were collected using a stratified two stage cluster sampling technique. In the first stage, a total of 539 enumeration areas (EAs) or clusters (138 in urban areas and 401 in rural areas), 540 EAs (145 urban and 395 rural), 624 clusters (187 in urban areas and 437 in rural areas), and 645 clusters (202 in urban areas and 443 in rural areas) were selected using systematic sampling with probability proportional to size, respectively the 2000, 2005, 2011 and 2016 EDHS surveys. At the second sampling stage, a systematic sample of households per EA was selected in all the regions to provide statistically reliable estimates of key demographic and health variables.

The EDHS used a questionnaire that was adapted from model survey tools developed for the DHS Program project. Mothers or caregivers provided all information related to children and mothers or caregivers through face-to-face interviews which were held at their homes. Water, Sanitation and Hygiene (WASH) indicators were also collected through face-to-face interviews and observation methods.

The EDHS collected data on children’s nutritional status by measuring the weight and height of under-fives in all sampled households. Weight was measured with an electronic mother-infant scale (SECA 878 flat) designed for mobile use. Height was measured with a measuring board (Shorr Board). Children younger than 24 months were measured lying down on the board (recumbent length), while standing height was measured for older children, in conformity with previous studies[29–32].

### Study variables

#### Outcome variables

The prevalence of stunting and wasting, defined by the World Health Organization (WHO), were the primary outcome variables of interest [33]. Height-for-age is a measure of linear growth retardation and cumulative growth deficits. Children, whose height-for-age Z-scores were below minus two standard deviations (-2 SD) from the median of the reference population, were considered short for their age (stunted) or chronically undernourished [33, 34].The weight-for-height index measures body mass in relation to body height or length and describes current nutritional status. Children, whose Z-scores below minus two standard deviations (-2 SD) from the median of the reference population, were considered thin (wasted) or acutely undernourished [33].

#### Exposure variables

The key exposure variables examined were all variables related to WASH, and specifically, sanitation facility (improved/unimproved), sources of drinking water (improved/unimproved), time to obtain drinking water (round trip) were classified as ‘water on premise’, ‘≤ 30 minutes round-trip fetching times’, ‘31–60 minutes round-trip fetching times’, ‘and > 60 minutes round-trip fetching times’, child stool disposal (safe/unsafe), and housing floor (improved/unimproved). A household floor was considered as improved only if households were without dirt floors. The World Health Organization (WHO)/ United Nations Children’s Fund (UNICEF)- Joint Monitoring Programme (JMP) for water improved supply and sanitation definition was taken into consideration in this study [35]. Unsafe disposal of children’s stool was defined as the disposal of faeces in any site other than a latrine, whereas other methods such as “child used latrine or latrine” and “put/rinsed into latrine or latrine” were considered as “safe disposal” [36] (Table 1).

**Table 1:** Exposure variable description and survey question

WASH factors	Type of variable & category	Survey question	Description
Toilet facility	Categorical data, categorised as “Improved”, “Unimproved” or “Open defecation”	What kind of toilet facility do members of your household usually use? (verify by observation)	Based on the WHO/UNICEF JMP definition, toilet facilities would be considered improved if they were any of the following types: flush/pour flush toilets to piped sewer systems, septic tanks, and pit latrines; ventilated improved pit (VIP) latrines; pit latrines with slabs; and composting toilets. Unimproved toilet facilities included: flush or pour-flush to elsewhere; pit latrine without a slab or open pit; bucket; hanging toilet og latrine. Other facilities, including households with no facility or use of bush/field, were considered open defecation.
Source of drinking water	Categorical data, categorised as “Improved”, or “Unimproved”	What is the main source of drinking water for members of your household?	Improved drinking water sources include piped water, public taps, standpipes, tube wells, boreholes, protected dug wells and springs, and rainwater. Other sources of drinking water are regarded as unimproved.
Child stool disposal	Binary data, categorised as “Safe” or “Unsafe”	The last time (NAME OF YOUNGEST CHILD living with the respondent) passed stool, what was done to dispose of the stool?	A child’s stool was considered to be disposed of “safely” when the child used a latrine/ toilet or child’s stool was put/rinsed into a toilet/latrine, whereas other methods were considered “unsafe”.
Household flooring	Binary data, categorised as “Improved” or “Unimproved”	Observe the main material of the floor of the dwelling. Record observation	Household floors are considered to be unimproved if it is natural floor (earth/sand, dung), rudimentary floor (wood planks, palm/bamboo), and finished floor (parquet or polished wood, vinyl or asphalt strips/plastic tile, ceramic tiles, cement, carpet) were considered as improved.
Time to obtain drinking water (round trip)	Categorical data, categorised as “On-premises”, “≤ 30 min round-trip fetching times”, “31–60 min round-trip fetching times”, and “ over 60 min round-trip fetching times”	How long does it take to go there, get water, and come back?	Time to obtain drinking water (round trip) was categorised as water on premises; up to 30 min, 31–60 min or over 60 min.

#### Confounders/control variables

As undernutrition results from a combination of factors, several control variables were considered in this study. We classified the control variables as child-related, parental-related, household-related, and community-related. As a result, the following factors were considered in the analysis. Child-related variables include: diarrhea, fever, symptoms of acute respiratory infection (ARI), sex, age (months), birth order, birth interval, size of child at birth (mother’s perceived baby size at birth), currently breastfeeding, early initiation of breastfeeding (children born in the past 2 years who started breastfeeding within one hour of birth), received all basic vaccination (i.e., child received a Bacillus Calmette–Guérin [BCG] vaccination against tuberculosis, 3 doses of Diphtheria, pertussis, and tetanus vaccine [DPT], ≥ 3 doses of polio vaccine [OPV], and 1 dose of measles vaccine). Parental-related factors included: mother’s age, mother’s educational level (no education, primary, secondary, and higher), mother’s occupation (not working, non-agriculture, or agriculture), antenatal care visits (ANC) (none, 1–3, or 4+), maternal body mass index (BMI), husband’s educational level, husband’s occupation (not working, non-agriculture, or agriculture), listening to the radio, and watching television. Household-level factors include: wealth index categorized (poor, middle, or rich) and household size (1–4 or ≥ 5). The wealth index is categorised into five wealth quintiles: ‘very poor’, ‘poor’, ‘middle’, ‘rich’ and ‘very rich. For this analysis, we re-coded the wealth index into three categories for adequate sampling in each category: ‘poor’ (poor and very poor), ‘middle’ and ‘rich’ (rich and very rich). Community-level factors include: ecological zone (tropical zone, subtropical zone, and cool zone), place of residence (urban and rural), and region (agrarian, pastoralist, and city-dweller).

### Statistical analysis

All statistical analyses were conducted using Stata™ software version 15.1 (Stata Corp, College Station, TX, USA). Descriptive statistics were used to describe the socio-demographic and economic characteristics of children included in the study. Differences in the two outcome variables “stunting” and “wasting” were presented across socio-demographic characteristics of interest using frequencies and percentages. A multilevel logistics regression analysis was performed using a stage modelling approach for each outcome (i.e., stunting and wasting). This means that each of the five-level factors (i.e., WASH, child-related factors, parental-related factors, household-related factors, and community-level factors) were examined using a series of multilevel logistic regression models, adjusting for selected potential confounders. A multilevel logistic regression model was used because of the nested structure of the EDHS data (i.e., individuals nested within households and households nested within clusters). Sampling weight was used during data analysis to adjust for non-proportional allocation of sample and possible differences in response rates across regions included in the survey. A detailed explanation of the weighting procedure has described in the EDHS methodology report [29–32]. Hierarchical multilevel models were run following the recommendations of a previous study that suggest complex hierarchical relationships of different determinants at different levels [37]. This approach allowed distal factors to be adequately investigated without interference from proximal factors [38]. A similar approach was also used to identify previous related literature [39].

In brief, a multilevel bivariable logistic regression model (*Model 0-* maximum model) was fitted with each explanatory variable to select candidates with p-value a < 0.20 for the stage multivariable models. Accordingly, *Model 1* incorporated WASH variables only. *Model 2* incorporated WASH plus child-related variables (all child-related explanatory variables with p-values < 0.2 from *Model 0* were entered into the *Model1*). *Model 3* incorporated WASH + child-related variables + parental-related factors (all parental-related variable with p-values < 0.2 from *Model 0* were entered into *Model 3*). *Model 4* incorporated WASH + child-related factors + parental-related factors + household-related factors (all household-related variables with p-values < 0.2 from *Model 0* were entered into the *model 4*). *Model 5* incorporated WASH + child-related variables + parental factors + household factors + community-level factors. *Model 6* was the final model that included only variables with a p-value < 0.2 from *Model 5*. Both crude odds ratio (COR) and adjusted odds ratio (AOR) ,along with 95% confidence intervals (CI), were used to estimate the strength of the association between explanatory and response variables.

## Results

### Summary of descriptive statistics

The background characteristics of children and prevalence of stunting and wasting across different background characteristics and covariates are presented in Table 2. In the current study, a total weighted sample of 33,744 and 33,763 under-five-year-old children was included to investigate child stunting and wasting, respectively. 51% of under-five children were males. 59% of children were older than twenty-four months. About one-third (33.9%) were from the rich categories. Nearly three-quarters (72.9%) of the mothers, and more than half of the husbands (54.2%) had no previous formal education. In this study, most children lived in rural (89.2%) and agrarian regions (54.4%). More than half (56.6%) of households practiced open defecation, 38.6% used unimproved sources of drinking water, and 78.9% practiced unsafe child stool disposal.

**Table 2:** Frequency distribution and reported prevalence of stunting and wasting among under-5 children by selected characteristics in Ethiopia, 2000–2016

Characteristics	Frequency	Weighted %	Stunting Prevalence (weighted %)	Wasting Prevalence (weighted %)
***WASH Facility***				
**Latrine facility**				
Improved	3,795	11.4	9.3	8.7
Unimproved	10,576	31.9	27.8	25.5
Open defecation	18,783	56.6	62.9	65.8
**Source of drinking water**				
Improved	20,356	61.4	35.6	37.5
Unimproved	12,791	38.6	64.4	62.5
**Child stool disposal**				
Safe	7,091	21.1	18.3	15.6
Unsafe	26,546	78.9	81.7	84.4
**Household flooring** ^**‡**^				
Improved	2,717	8.1	4.8	5.4
Unimproved	31,736	91.9	95.2	94.6
**Time to get water source**				
On premise	1,770	5.3	2.9	3.8
<=30 min	19,545	58.3	60.1	57.7
31–60 min	6,894	20.6	21.1	21.3
> 60 min	5,327	15.9	15.9	17.2
**Household drinking water service**				
Basic drinking water service	8,549	25.3	23.1	24.4
Limited drinking water service	4,036	11.9	11.3	12.1
Poor drinking water service	21,158	62.7	65.6	63.5
**Combined sanitation facility**				
Improved Water + Improved Sanitation	2,342	7.1	5.1	4.6
Either one improved	11,901	35.9	34.6	37.2
Unimproved Water + Unimproved Sanitation	18,904	57.0	60.3	58.2
***Child Factors***				
***Childhood infections***				
**Diarrhea**				
Yes	5,759	17.1	18.7	24.8
No	27,938	82.9	81.3	75.2
**Fever**				
Yes	6,853	20.3	21.4	28.1
No	26,841	79.7	78.6	71.9
**ARI**				
Yes	1,354	4.0	3.6	4.8
No	32,389	96.0	96.4	95.2
**Sex**				
Male	17,172	50.9	52.9	55.6
Female	16,571	49.1	47.1	44.4
**Age (months)**				
0–5	3,436	10.2	3.4	13.4
6–11	3,565	10.6	5.7	16.7
12–17	3,561	10.5	9.7	16.3
18–23	3,001	8.9	10.5	10.8
≥ 24 to 59 months	20,179	59.8	70.7	42.7
**Birth order**				
Firstborn	6,005	17.8	16.8	15.5
2–4	14,547	43.1	42.4	42.0
5 or higher	13,190	39.1	40.8	42.5
**Birth interval**				
< 33 months	23,276	69.0	67.6	67.6
≥ 33 months	10,467	31.0	32.4	32.4
**Size of a child at birth**				
Larger	10,431	31.0	28.5	29.9
Average	13,236	39.3	38.1	36.9
Small	9,979	29.7	33.4	38.2
**Currently breastfeeding**				
Yes	25,031	74.2	73.1	81.6
No	8,712	25.8	26.9	18.4
**Early initiation of breastfeeding**				
Yes	14,143	54.4	52.0	52.2
No	11,848	45.6	48.0	47.8
**Received measles**				
Yes	10,768	36.8	39.5	29.4
No	18,501	63.2	60.5	70.6
**Basic vaccine**				
Yes	5,608	19.4	20.3	15.1
No	23,250	80.6	79.7	84.9
***Parental factors***				
**Mother’s age**				
< 18	278	0.8	0.8	1.0
18–24	7,818	23.2	22.0	23.8
25–34	17,123	50.7	50.8	48.4
≥ 35	8,524	25.3	26.4	26.4
**Mother’s education**				
No education	24,596	72.9	77.6	79.3
Primary	7,398	21.9	19.5	17.7
Secondary	1,353	4.0	2.4	2.3
Higher	396	1.2	0.5	0.7
**Mother’s occupation**				
Not working	16,401	48.8	46.1	48.5
Non agriculture	7,073	21.0	19.8	18.0
Agriculture	10,164	30.2	34.1	33.5
**ANC Visit**				
None	13,214	57.1	63.4	62.4
1–3	5,323	23.0	21.4	22.2
4+	4,587	19.9	15.2	15.4
**Maternal BMI (**kg/m^2^**)**				
< 18.5	7,053	21.0	22.4	30.4
18.5 to 24.9	25,082	74.8	74.8	67.5
25 +	1,411	4.2	2.8	2.1
**Husband’s education**				
No education	17,834	54.2	59.5	62.6
Primary	11,619	35.3	33.0	30.3
Secondary	2,540	7.7	6.1	5.4
Higher	886	2.7	1.4	1.7
**Listening to radio**				
Not at all	22,157	65.7	69.0	71.8
Yes	11,577	34.3	31.0	28.2
**Watching television**				
Not at all	27,820	82.5	85.9	87.8
Yes	5,901	17.5	14.1	12.2
***Household Factors***				
**Wealth index**				
Poor	10,848	45.3	49.9	55.7
Middle	4,998	20.9	21.2	20.9
Rich	8,087	33.8	28.9	23.4
**Household Size**				
1–4	8,097	24.0	22.9	23.3
≥ 5	25,647	76.0	77.1	76.7
***Community*** ***level factors***				
**Residence**				
Urban	3,639	10.8	7.9	7.3
Rural	30,104	89.2	92.1	92.7
**Region**				
Agrarian	18,365	54.4	58.6	56.8
Pastoralist	14,591	43.2	40.1	41.9
City	787	2.4	1.3	1.3
**Ecological Zone (meters in elevation )** ^**@**^ **(n = 34,058)**				
< 1500	3,601	15.1	13.3	19.0
1500–2500	16,833	70.3	70.2	68.4
> 2500	3,498	14.6	16.5	12.6

‡: In this analysis rudimentary and finished floor types are considered improved (households without dirt floor), while only natural flooring is considered sub-optimal (households with dirt floor). ARI: symptoms of acute respiratory infection

@ *Kolla* (Tropical zone) - is below 1500 m in elevation; *Woina dega* (Subtropical zone) - includes the highlands areas of 1500–2500 m in elevation; *Dega* (Cool zone) - is above 2500 m in elevation

### Prevalence of stunting and wasting

The overall prevalences of stunting and wasting were found to be 47.29% (95% CI: 46.75, 47.82%) and 10.98% (95% CI: 10.65, 11.32%), respectively. The prevalence of stunting among males was higher than females (52.9%; 47.1%), and similarly for wasting (55.6%; 44.4%). There was a higher burden of stunting in rural areas (92.1%) than in urban areas (7.9%). Children in households practising open defecation had a higher prevalence of stunting (62.9%) and wasting (65.8%) compared to their counterparts who did not practise open defecation (Table 2).

The prevalence of stunting and wasting by other WASH, child, and parental characteristics is shown in Table 3. In the multilevel bivariable binary logistic regression, we assessed the unadjusted or crude relationship between WASH and the prevalence of stunting and wasting among children (**Additional File 1 and 2).** The crude association revealed that the children from households with unimproved WASH facilities faced comparatively higher occurrences of stunting and wasting.

**Table 3:** **Odds ratio estimates on the association between stunting and wasting and other factors on the prevalence of stunting and wasting among under-5 children, Ethiopia, 2000–2016**

Characteristics	Stunting (OR, 95%CI)	p-value	Wasting (OR, 95%CI)	p-value
***WASH Facility***				
**Latrine facility**				
Improved	1		1	
Unimproved	1.61 (1.49,1.74)	p < 0.001	1.11 (0.98,1.26)	0.087
Open defecation	2.13 (1.98,2.29)	p < 0.001	1.78 (1.60,1.98)	p < 0.001
**Source of drinking water**				
Improved	1		1	
Unimproved	1.39 (1.32,1.46)	p < 0.001	1.17 (1.09,1.25)	p < 0.001
**Child stool disposal**				
Safe	1		1	
Unsafe	1.55 (1.46,1.64)	p < 0.001	1.50 (1.37,1.65)	p < 0.001
**Household flooring**				
Improved	1		1	
Unimproved	2.82 (2.60,3.05)	p < 0.001	1.87 (1.65,2.11)	p < 0.001
**Time to get a water source**				
On-premise	1		1	
≤ 30 min	2.80 (2.53,3.09)	p < 0.001	1.59 (1.37, 1.86)	p < 0.001
31–60 min	2.82 (2.53, 3.14)	p < 0.001	1.78 (1.51,2.09)	p < 0.001
> 60 min	2.76 (2.47, 3.08)	p < 0.001	2.06 (1.74,2.43)	p < 0.001
**Household drinking water service**				
Basic drinking water service	1			
Limited drinking water service	1.25 (1.15,1.36)	p < 0.001	1.26 (1.12,1.43)	p < 0.001
Poor drinking water service	1.42 (1.34,1.49)	p < 0.001	1.22 (1.12,1.33)	p < 0.001
**Combined sanitation facility**				
Improved Water + Improved Sanitation	1		1	
Either one improved	2.19 (2.01,2.39)	p < 0.001	1.82 (1.58,2.09)	p < 0.001
Unimproved Water + Unimproved Sanitation	2.57 (2.36,2.79)	p < 0.001	1.88 (1.64,2.15)	p < 0.001
***Child Factors***				
***Childhood infections***				
**Diarrhea**				
Yes	1.25 (1.17,1.33)	p < 0.001	1.64 (1.51,1.79)	p < 0.001
No	1		1	
**Fever**				
Yes	1.19 (1.13,1.26)	p < 0.001	1.56 (1.44,1.69)	p < 0.001
No	1			
**ARI**				
Yes	0.92 (0.82,1.04)	0.206	1.29 (1.09,1.53)	0.002
No	1		1	
**Sex**				
Male	1		1	
Female	0.89 (0.84,0.93)	p < 0.001	0.82 (0.76,0.87)	p < 0.001
**Age (months)**				
< 12	1		1	
12–23	4.19 (3.85,4.55)	p < 0.001	0.89 (0.81,0.98)	0.019
≥ 24 to 59 months	5.43 (5.05,5.83)	p < 0.001	0.45 (0.42,0.49)	p < 0.001
**Birth order**				
First born	1		1	
2–4	1.13 (1.07,1.21)	p < 0.001	1.16 (1.05,1.28)	0.003
5 or higher	1.34 (1.25,1.43)	p < 0.001	1.35 (1.22,1.49)	p < 0.001
**Birth interval**				
< 33 months	1		1	
≥ 33 months	1.15 (1.10,1.22)	p < 0.001	1.01 (0.94,1.09)	0.695
**Size of child at birth**				
Larger	1		1	
Average	1.11 (1.05,1.17)	p < 0.001	1.19 (1.09,1.30)	p < 0.001
Small	1.46 (1.37,1.55)	p < 0.001	1.79 (1.63,1.95)	p < 0.001
**Currently breastfeeding**				
Yes	1		1	
No	1.17 (1.11,1.23)	p < 0.001	0.69 (0.63,0.74)	p < 0.001
**Early initiation of breastfeeding**				
Yes	1		1	
No	1.14 (1.08,1.20)	p < 0.001	1.04 (0.96,1.12)	0.314
**Received measles**				
Yes	1		1	
No	0.81 (0.77,0.85)	p < 0.001	1.61 (1.49,1.74)	p < 0.001
**Basic vaccine**				
Yes	1		1	
No	0.94 (0.88,0.99)	0.033	1.61 (1.46,1.78)	p < 0.001
***Parental factors***				
**Mother’s age**				
< 18	1		1	
18–24	1.37 (1.04,1.79)	0.023	0.71 (0.51,1.01)	0.051
25–34	1.55 (1.18,2.03)	0.001	0.64 (0.46,090)	0.011
≥ 35	1.72 (1.31,2.26)	p < 0.001	0.69 (0.49,0.98)	0.038
**Mother’s education**				
No education	5.56 (4.36,7.09)	p < 0.001	2.70 (1.87,3.90)	p < 0.001
Primary	3.79 (2.96, 4.85)	p < 0.001	1.91 (1.31, 2.78)	0.001
Secondary	1.85 (1.42, 2.41)	p < 0.001	1.24 (0.82, 1.87)	0.306
Higher	1		1	
**Mother’s occupation**				
Not working	1		1	
Non agriculture	0.95 (0.89,1.01)	0.110	0.74 (0.67,0.81)	p < 0.001
Agriculture	1.54 (1.46,1.63)	p < 0.001	1.02 (0.94, 1.11)	0.541
**ANC Visit**				
None	1		1	
1–3	0.71 (0.66,0.76)	p < 0.001	0.84 (0.77,0.93)	p < 0.001
4+	0.48 (0.44,0.52)	p < 0.001	0.52 (0.47,0.58)	p < 0.001
**Maternal BMI (**kg/m^2^)				
< 18.5	1		1	
18.5 to 24.9	0.89 (0.84,0.94)	p < 0.001	0.55 (0.51,0.59)	p < 0.001
25 +	0.37 (0.32,0.41)	p < 0.001	0.26 (0.21,0.32)	p < 0.001
**Husband’s education**				
No education	1		1	
Primary	3.64 (3.17,4.19)	p < 0.001	1.82 (1.49,2.23)	p < 0.001
Secondary	2.75 (2.38,3.18)	p < 0.001	1.24 (1.01,1.54)	0.037
Higher	1.69 (1.44,1.97)	p < 0.001	1.18 (0.94,1.49)	0.148
**Listening to radio**				
Not at all	1		1	
Yes	0.75 (0.72,0.79)	p < 0.001	0.66 (0.61,0.71)	p < 0.001
**Watching television**				
Not at all	1		1	
Yes	0.52 (0.49,0.55)	p < 0.001	0.53 (0.47,0.58)	p < 0.001
***Household factors***				
**Wealth index**				
Poor	1.71 (1.59,1.81)	p < 0.001	1.95 (1.76,2.16)	p < 0.001
Middle	1.52 (1.39,1.65)	p < 0.001	1.52 (1.33,1.74)	p < 0.001
Rich	1		1	
**Household Size**				
1–4	0.86 (0.82,0.91)	p < 0.001	0.89 (0.82,0.97)	0.006
≥ 5	1		1	
***Community-Level Factors***				
**Residence**				
Urban	1		1	
Rural	2.29 (2.14,2.46)	p < 0.001	1.61 (1.45,1.80)	p < 0.001
**Region**				
Agrarian	1.91 (1.77,2.05)	p < 0.001	1.55 (1.38,1.74)	p < 0.001
Pastoralist	1.78 (1.63,1.93)	p < 0.001	1.55 (1.37,1.76)	p < 0.001
City	1		1	
**Ecological Zone (meters in elevation)**				
< 1500	0.65 (0.58,0.73)	p < 0.001	2.01 (1.68,2.41)	p < 0.001
1500–2500	0.73 (0.66,0.82)	p < 0.001	1.23 (1.03,1.48)	p < 0.001
> 2500	1		1	

AOR: Adjusted Odds Ratio; COR: Crude Odds Ratio

### WASH factors associated with stunting

WASH factors associated with stunting included latrine facilities, sources of drinking water, and household flooring. Children from households having unimproved latrine facilities [AOR: 1.20, 95% CI: (1.05, 1.39)], practising open defecation [AOR: 1.29, 95% CI: (1.11, 1.51)], and living in households with dirt floors [AOR: 1.32, 95% CI: (1.12, 1.57)] were more likely to be stunted. Those having unimproved drinking water sources were significantly less likely to be stunted [AOR: 0.91, 95% CI: (0.83, 0.99)] (Table 4).

**Table 4:** Adjusted odds ratio estimates on the effects of WASH and other factors on the prevalence of stunting among under-5 children, Ethiopia: 2000–2016

Characteristics	Model 1 (AOR, 95%CI)	p-value	Model 2 (AOR, 95%CI)	p-value	Model 3 (AOR, 95%CI)	p-value	Model 4 (AOR, 95%CI)	p-value	Model 5 (AOR, 95%CI)	p-value	Model 6^#^ (AOR, 95%CI)	p-value
***WASH factors*** ^**a**^												
**Latrine facility**												
Improved	1		1		1		1		1		1	
Unimproved	1.08 (0.98,1.18)	0.087	1.12 (1.01,1.26)	0.048	1.03 (0.91,1.15)	0.670	1.25 (1.08,1.45)	0.002	1.21 (1.05,1.39)	0.009	**1.20 (1.05,1.39)**	0.009
Open defecation	1.34 (1.23,1.47)	p < 0.001	1.39 (1.24,1.56)	p < 0.001	1.21 (1.08,1.37)	0.002	1.30 (1.11,1.52)	0.001	1.29 (1.11,1.51)	0.001	**1.29 (1.11,1.51)**	0.001
**Source of drinking water**												
Improved	1		1		1		1		1		1	
Unimproved	1.04 (0.99,1.10)	0.100	1.04 (0.97,1.12)	0.265	1.01 (0.93,1.08)	0.879	0.91 (0.84,0.99)	0.042	0.92 (0.84,1.01)	0.06	**0.91 (0.83,0.99)**	0.041
**Child stool disposal**												
Safe	1		1		1		1		1		1	
Unsafe	1.08 (1.01,1.16)	0.022	1.08 (0.98,1.18)	0.092	0.98 (0.89,1.08)	0.782	0.94 (0.85,1.05)	0.302	0.94 (0.85,1.05)	0.260	0.94 (0.85,1.04)	0.282
**Household flooring**												
Improved	1		1		1		1		1		1	
Unimproved	1.85 (1.67,2.05)	p < 0.001	1.79(1.57,2.04)	p < 0.001	1.37 (1.19,1.58)	p < 0.001	1.38 (1.17,1.62)	p < 0.001	1.31 (1.10,1.55)	0.002	**1.32 (1.12,1.57)**	0.001
**Time to get water source**												
On-premise	1		1		1		1		1		1	
≤ 30 min	1.67 (1.49,1.88)	p < 0.001	1.55 (1.33,1.82)	p < 0.001	1.28 (1.09,1.50)	0.003	1.16 (0.97,1.37)	0.097	1.09 (0.91,1.30)	0.335	1.09 (0.91,1.29)	0.338
31–60 min	1.64 (1.45,1.86)	p < 0.001	1.49 (1.27,1.78)	p < 0.001	1.21 (1.02,1.44)	0.030	1.09 (0.90,1.32)	0.356	1.03 (0.85,1.24)	0.789	1.02 (0.84,1.24)	0.795
> 60 min	1.56 (1.37,1.77)	p < 0.001	1.47 (1.24,1.74)	p < 0.001	1.18 (0.99,1.41)	0.069	1.08 (0.89,1.31)	0.448	1.04 (0.86,1.27)	0.665	1.05 (0.86,1.27)	0.618
***Child Factors***												
***Childhood infections***												
**Diarrhea**												
Yes			1.22 (1.13,1.33)	p < 0.001	1.19 (1.09,1.30)	p < 0.001	1.14 (1.02,1.27)	0.018	1.14 (1.02,1.27)	0.018	1.16 (1.05,1.28)	0.003
No			1		1		1		1		1	
**Fever**												
Yes			1.14 (1.06,1.23)	p < 0.001	1.14 (1.06,1.24)	0.001	1.07 (0.96,1.19)	0.187	1.06 (0.96,1.18)	0.223		
No			1		1				1			
**ARI**												
Yes			0.79 (0.67,0.94)	0.008	0.80 (0.67,0.95)	0.010	0.91 (0.76,1.08)	0.275				
No			1		1		1					
**Sex**												
Male			1		1		1		1		1	
Female			0.81 (0.76,0.86)	p < 0.001	0.79 (0.74,0.85)	p < 0.001	0.79 (0.73,0.85)	p < 0.001	0.79 (0.73,0.85)	p < 0.001	0.79 (0.72,0.85)	p < 0.001
**Age (months)**												
< 12			1		1		1		1		1	
12–23			3.54 (3.09,4.06)	p < 0.001	3.66 (3.13,4.29)	p < 0.001	3.19 (2.61,3.90)	p < 0.001	3.17 (2.60,3.85)	p < 0.001	3.16 (2.59,3.84)	p < 0.001
≥ 24 to 59 months			6.83 (5.93,7.87)	p < 0.001	7.51 (6.29,8.96)	p < 0.001	6.63 (5.32,8.27)	p < 0.001	6.52 (5.25,8.09)	p < 0.001	6.47 (5.21,8.02)	p < 0.001
**Birth order**												
Firstborn			1		1		1		1		1	
2–4			1.04 (0.95,1.14)	0.382	0.90 (0.81,1.01)	0.076	0.85 (0.75,0.96)	0.011	0.86 (0.76,0.97)	0.016	0.88 (0.78,0.98)	0.032
5 or higher			1.17 (1.05,1.30)	0.004	0.95 (0.83,1.09)	0.513	0.81 (0.70,0.94)	0.005	0.81 (0.70,0.94)	0.004	0.85 (0.75,0.96)	0.009
**Birth interval**												
< 33 months			1		1							
≥ 33 months			0.91 (0.83,0.98)	0.023	0.91 (0.78,1.06)	0.255						
**Size of child at birth**												
Larger			1		1		1		1		1	
Average			1.21 (1.12,1.31)	p < 0.001	1.18 (1.09,1.28)	p < 0.001	1.23 (1.12,1.35)	p < 0.001	1.22 (1.11,1.34)	p < 0.001	1.22 (1.11,1.34)	p < 0.001
Small			1.68 (1.55,1.82)	p < 0.001	1.63 (1.49,1.78)	p < 0.001	1.65 (1.48,1.83)	p < 0.001	1.64 (1.48,1.81)	p < 0.001	1.64 (1.48,1.82)	p < 0.001
**Currently breast feeding**												
Yes			1		1		1		1		1	
No			0.70 (0.65,0.76)	p < 0.001	0.63 (0.58,0.69)	p < 0.001	0.61 (0.55,0.67)	p < 0.001	0.62 (0.55,0.68)	p < 0.001	0.62 (0.55,0.68)	p < 0.001
**Early initiation of breastfeeding**												
Yes			1		1		1					
No			1.04 (0.98,1.11)	0.159	1.05 (0.98,1.12)	0.153	1.02 (0.93,1.10)	0.683				
**Received measles**												
Yes			1									
No			1.04 (0.95, 1.14)	0.359								
**Basic vaccine**												
Yes			1									
No			1.01 (0.91,1.11)	0.943								
***Parental factors***												
**Mother’s age**												
< 18					1							
18–24					0.80 (0.55,1.14)	0.228						
25–34					0.81 (0.56,1.16)	0.252						
≥ 35					0.77 (0.51,1.15)	0.201						
**Mother’s education**												
No education					1.60 (1.12, 2.29)	0.011	1.41 (0.96,2.04)	0.074	1.51 (1.04,2.19)	0.031	1.54 (1.06,2.24)	0.022
Primary					1.39 (0.97,1.98)	0.068	1.25 (0.87,1.81)	0.219	1.35 (0.94,1.96)	0.100	1.38 (0.96,1.99)	0.080
Secondary					1.20 (0.83,1.73)	0.316	1.12 (0.77,1.63)	0.539	1.20 (0.83,1.75)	0.336	1.21 (0.83,1.77)	0.305
Higher					1		1		1		1	
**Mother’s occupation**												
Not working					1		1		1		1	
Non agriculture					1.02 (0.94,1.12)	0.595	0.99 (0.89,1.11)	0.975	1.01 (0.90,1.11)	0.943	1.01 (0.90,1.11)	0.958
Agriculture					1.18 (1.09,1.28)	p < 0.001	1.10 (0.99,1.23)	0.058	1.07 (0.96,1.19)	0.186	1.06 (0.96,1.18)	0.235
**ANC Visit**												
None					1		1		1		1	
1–3					0.87 (0.80,0.95)	0.002	0.93 (0.85,1.04)	0.210	0.93 (0.84,1.03)	0.151	0.93 (0.84,1.02)	0.136
4+					0.72 (0.65,0.79)	p < 0.001	0.74 (0.66,0.83)	p < 0.001	0.75 (0.67,0.83)	p < 0.001	0.74 (0.66,0.83)	p < 0.001
**Maternal BMI (**kg/m^2^)												
< 18.5					1		1		1		1	
18.5 to 24.9					0.95 (0.88,1.02)	0.166	0.93 (0.85,1.01)	0.105	0.92 (0.84,1.02)	0.073	0.92 (0.84,1.01)	0.072
25 +					0.81 (0.67,0.96)	0.018	0.79 (0.65,0.96)	0.017	0.79 (0.66,0.97)	0.023	0.79 (0.65,0.96)	0.020
**Husband’s education**												
No education					1.45 (1.15,1.83)	0.001	1.51 (1.17,1.94)	0.001	1.50 (1.17,1.92)	0.001	1.50 (1.17,1.92)	0.001
Primary					1.32 (1.05,1.65)	0.017	1.39 (1.09,1.78)	0.008	1.36 (1.07,1.74)	0.011	1.37 (1.07,1.74)	0.011
Secondary					1.09 (0.86,1.37)	0.453	1.13 (0.88,1.46)	0.336	1.15 (0.89,1.48)	0.260	1.15 (0.89,1.48)	0.264
Higher					1		1		1		1	
**Husband’s occupation**												
Not working					0.69 (0.57,0.84)	p < 0.001	0.75 (0.61,0.93)	0.009	0.75 (0.61,0.92)	0.006	0.75 (0.61,0.93)	0.008
Agriculture					1.04 (0.94,1.15)	0.410	1.10 (0.98,1.24)	0.088	1.07 (0.95,1.20)	0.283	1.07 (0.95,1.21)	0.241
Non agriculture					1		1		1		1	
**Listening to radio**												
Not at all					1							
Yes					0.98 (0.91,1.06)	0.669						
**Watching television**												
Not at all					1		1		1			
Yes					0.87 (0.78,0.97)	0.011	0.92 (0.82,1.03)	0.147	0.93 (0.83,1.04)	0.210		
***Household factors***												
**Wealth index**												
Poor							1.17 (1.04,1.31)	0.009	1.19 (1.06,1.31)	0.003	1.20 (1.07,1.35)	0.002
Middle							0.99 (0.87,1.13)	0.936	1.01 (0.88,1.15)	0.827	1.01 (0.89,1.15)	0.828
Rich							1		1		1	
**Household Size**												
1–4							0.93 (0.84,1.03)	0.187	0.94 (0.85,1.04)	0.240		
≥ 5							1		1			
***Community Level Factors***												
**Residence**												
Urban									1		1	
Rural									1.13 (0.94,1.35)	0.181	1.15 (0.96,1.37)	0.123
**Region**												
Agrarian									1.11 (0.96,1.28)	0.176	1.12 (0.97,1.29)	0.124
Pastoralist									1.08 (0.92,1.27)	0.320	1.09 (0.93,1.28)	0.276
City administrations									1		1	
**Ecological Zone**												
< 1500									0.67 (0.58,0.78)	p < 0.001	0.67 (0.58,0.78)	p < 0.001
1500–2500									0.75 (0.65,0.87)	p < 0.001	0.75 (0.65,0.87)	p < 0.001
> 2500									1		1	

AOR (Adjusted Odds Ratio); COR (Crude Odds Ratio), WASH (Water, Sanitation, and Hygiene), LL: Log-likelihood

Model 0: Empty model (-LL: -21703.816), ICC(%): 2.93, AIC: 43411.63, BIC: 43428.36

Model 1: WASH variables only (-LL: -19976.364), ICC(%): 2.84, AIC: 39972.73, BIC: 40055.76

Model 2: WASH + Child related factors (from model 0 with p < 0.20) (-LL: -12070.816)

Model 3: WASH + Child related factors (from model 2 with p < 0.20) + Parental factors (from model 0 with p < 0.20) (-LL: -10882.449)

Model 4: WASH + Child related factors (p < 0.20) + Parental factors (from model 0 with p < 0.20) + Household factors (-LL: -7718.3869)

Model 5: WASH + Child related factors (p < 0.20) + Parental factors (from model 0 with p < 0.20) + Household factors (from model 4 with p < 0.2) + Community level factors (-LL: --7816.6514), ICC (%): 3.08; AIC: 15723.3, BIC: 16061.63

Model 6: (final model): The final model includes only variables with a p < 0.2 from model 5. (-LL: --7830.3954), ICC (%): 3.0; AIC: 15744.79, BIC: 16060.62

^a^Variables such as household drinking water service and combined sanitation facility were removed by the model due to multicollinearity

# WASH factors significant associated with stunting (p < 0.05)

In the final model, being female [AOR: 0.79, 95% CI: (0.72, 0.85)], birth order 2nd to 4th [AOR: 0.88, 95% CI: (0.78–0.98)], and birth order 5th or higher [AOR: 0.85, 95% CI: (0.75–0.96)] were less likely to be stunted. Children aged 12–23 months [AOR: 3.16; 95%: (2.59, 3.84)], aged ≥ 24 months [AOR: 6.47, 95% CI: (5.21–8.02)], average birth size [AOR:1.22, 95% CI: (1.11,1.34)], small size at birth [AOR:1.64, 95% CI: (1.48,1.82)], lack of maternal education [AOR: 1.54, 95% CI: (1.06,2.24)], lack of father education [AOR: 1.50, 95% CI: (1.17,1.92)], husband having primary education [AOR: 1.37,95% CI: (1.07,1.74)] were associated with increased odds of being stunted. Husbands being unemployed [AOR: 0.75, 95% CI: (0.61, 0.93)], and mothers with BMI ≥ 25 kg/m^2^ [AOR: 0.79, 95% CI: (0.65, 0.96)] were significantly associated with lower odds of being stunted. Children from poor households [AOR: 1.20, 95% CI: (1.07,1.35)] had higher odds of being stunted compared with children from the richest households. At the community level, children who lived in tropical [AOR: 0.67, 95% CI: (0.58,0.78)] and lived subtropical ecological zone [AOR: 0.75, 95% CI: (0.65,0.87)] were associated with lower odds of being stunted (Table 4).

### WASH factors associated with wasting

We observed no evidence of an association between improved sanitation, safe disposal of a child’s stool, or improved household flooring and child wasting. Having unimproved drinking water sources was associated with lower odds of being wasted [AOR: 0.83, 95% CI: (0.73,0.93)]. Control variables associated with wasting included having diarrhea [AOR: 1.27, 95%CI: (1.11, 1.45)], having fever [AOR: 1.24, 95% CI: (1.09, 1.41)], birth order 5th or higher [AOR: 1.28, 95% CI: (1.09, 1.50)], and small size at birth [AOR: 1.58, 95% CI: (1.40, 1.82)] were associated with elevated odds of being wasted. Children from poor households [AOR: 1.40, 95% CI: (1.18, 1.66)] and those from middle households [AOR: 1.27, 95% CI: (1.05, 1.53)] reported higher odds of being wasted than those children from richest households. Being female [AOR: 0.73, 95% CI: (0.65,0.81)], age greater than 24 months [AOR: 0.62, 95% CI: (0.50,0.83)], having four and more ANC visits [AOR: 0.74, 95% CI: (0.64,0.87)], normal maternal BMI [AOR: 0.65, 95% CI: (0.58,0.73)], women classified as ‘overweight/obese’ [AOR: 0.39, 95% CI: (0.28,0.52)], and watching television [AOR: 0.71, 95% CI: (0.61,0.84)] were associated with lower odds of being wasted. At the community level, rural dwellers [AOR: 0.58, 95% CI: (0.46, 0.73)], and children who lived in tropical ecological zone [AOR: 1.61, 95% CI: (1.30, 1.99)] reported higher odds of being wasted (Table 5).

**Table 5:** Adjusted odd ratio estimates on the effects of WASH and other factors on prevalence of wasting among under-5 children, Ethiopia: 2000–2016

Characteristics	Model 1 (AOR, 95%CI)	p-value	Model 2 (AOR, 95%CI)	p-value	Model 3 (AOR, 95%CI)	p-value	Model 4 (AOR, 95%CI)	p-value	Model 5 (AOR, 95%CI)	p-value	Model 6^#^ (AOR, 95%CI)	p-value
***WASH factors*** ^**a**^												
**Latrine facility**												
Improved	1		1		1		1		1		1	
Unimproved	0.93 (0.81,1.07)	0.330	1.12 (1.01,1.24)	0.024	1.05 (0.92,1.18)	0.471	1.26 (1.09,1.45)	0.001	1.03 (0.83,1.26)	0.792	1.02 (0.83,1.24)	0.873
Open defecation	1.39 (1.21,1.59)	p < 0.001	1.31 (1.19,1.45)	p < 0.001	1.21 (1.06,1.37)	0.004	1.30 (1.12,1.52)	0.001	1.15 (0.93,1.42)	0.206	1.15 (0.93, 1.42)	0.182
**Source of drinking water**												
Improved	1		1		1		1		1		1	
Unimproved	0.98 (0.91,1.06)	0.635	1.05 (0.99,1.12)	0.089	1.01 (0.94,1.09)	0.696	0.91 (0.84,0.99)	0.039	0.84 (0.75,0.94)	0.004	**0.83 (0.73,0.93)**	0.002
**Child stool disposal**												
Safe	1		1		1		1		1		1	
Unsafe	1.07 (0.96,1.19)	0.208	1.14 (1.05,1.23)	0.002	1.05 (0.95,1.16)	0.310	0.96 (0.86,1.06)	0.404	1.09 (0.94,1.26)	0.233	1.09 (0.94,1.25)	0.239
**Household flooring**												
Improved	1		1		1		1`		1		1	
Unimproved	1.40 (1.19,1.64)	p < 0.001	1.78 (1.58,2.00)	p < 0.001	1.37 (1.18,1.59)	p < 0.001	1.36 (1.15,1.60)	p < 0.001	0.99 (0.78,1.26)	0.948	0.99 (0.78,1.25)	0.957
**Time to get water source**												
On-premise	1		1		1		1		1		1	
≤ 30 min	1.11 (0.93,1.32)	0.246	1.66 (1.45,1.91)	p < 0.001	1.26 (1.06,1.50)	0.006	1.14 (0.97,1.36)	0.114	1.14 (0.89,1.46)	0.313	1.11 (0.86,1.42)	0.412
31–60 min	1.20 (0.99,1.45)	0.059	1.55 (1.34,1.81)	p < 0.001	1.19 (0.99,1.43)	0.054	1.07 (0.89,1.29)	0.444	1.15 (0.88,1.50)	0.313	1.14 (0.87,1.48)	0.336
> 60 min	1.37 (1.13,1.65)	0.001	1.47 (1.26,1.71)	p < 0.001	1.18 (0.98,1.42)	0.083	1.07 (0.88,1.29)	0.483	1.24 (0.95,1.62)	0.116	1.24 (0.95,1.62)	0.112
***Child factors***												
***Childhood infections***												
**Diarrhea**												
Yes			1.28(1.19,1.38)	p < 0.001	1.19 (1.09,1.31)	p < 0.001	1.34 (1.02,1.27)	0.020	1.26 (1.09,1.44)	0.001	1.27 (1.11,1.45)	p < 0.001
No			1		1		1		1			
**Fever**												
Yes			1.17 (1.09,1.26)	p < 0.001	1.14 (1.05,1.25)	0.001	1.08 (0.97,1.20)	0.135	1.25 (1.09,1.42)	0.001	1.24 (1.09,1.41)	0.001
No			1				1		1		1	
**ARI**												
Yes			0.83 (0.72,0.96)	0.014	0.79 (0.67,0.95)	0.011	0.91 (0.77,1.08)	0.304				
No			1		1		1					
**Sex**												
Male			1		1		1		1		1	
Female			0.84 (0.79,0.88)	p < 0.001	0.77 (0.72,0.83)	p < 0.001	0.78 (0.73,0.85)	p < 0.001	0.73 (0.65,0.81)	p < 0.001	0.73 (0.65,0.81)	p < 0.001
**Age (months)**												
< 12			1		1		1		1		1	
12–23			3.60 (3.17,4.08)	p < 0.001	3.57 (3.05,4.19)	p < 0.001	3.17 (2.60,3.85)	p < 0.001	1.07 (0.85,1.35)	0.548	1.08 (0.86, 1.36)	0.508
≥ 24 to 59 months			5.57 (4.91,6.31)	p < 0.001	7.38 (6.17,8.82)	p < 0.001	6.56 (5.28,8,14)	p < 0.001	0.67 (0.51,0.87)	0.003	0.65 (0.50, 0.83)	0.001
**Birth order**												
Firstborn			1		1			1	1		1	
2–4			1.05 (0.97,1.13)	0.202	0.88 (0.79,0.98)	0.032	0.85 (0.76,0.97)	0.013	1.07 (0.91,1.25)	0.392	1.10 (0.94,1.29)	0.214
5 or higher			1.15 (1.06,1.24)	p < 0.001	0.92 (0.79,1.05)	0.214	0.82 (0.71,0.94)	0.005	1.21 (1.02,1.43)	0.024	1.28 (1.09,1.50)	0.002
**Size of child at birth**												
Larger			1		1		1		1		1	
Average			1.22 (1.14,1.31)	p < 0.001	1.19 (1.08,1.29)	p < 0.001	1.22 (1.10,1.34)	p < 0.001	1.14 (0.99,1.29)	0.065	1.13 (0.99,1.29)	0.055
Small			1.63 (1.52,1.75)	p < 0.001	1.62 (1.48,1.77)	p < 0.001	1.64 (1.47,1.82)	p < 0.001	1.56 (1.36,1.79)	p < 0.001	1.58 (1.40,1.82)	p < 0.001
**Currently breast feeding**												
Yes			1		1		1		1			
No			0.83 (0.78,0.89)	p < 0.001	0.67 (0.61,0.73)	p < 0.001	0.61 (0.54,0.67)	p < 0.001	0.91 (0.78,1.06)	0.238		
**Received measles**												
Yes			1		1							
No			1.06 (0.98,1.15)	0.116	0.96 (0.89,1.04)	0.393						
**Basic vaccine**												
Yes			1									
No			0.97 (0.89,1.06)	0.569								
***Parental factors***												
**Mother’s age**												
< 18					1							
18–24					0.79 (0.55,1.14)	0.206						
25–34					0.82 (0.56,1.19)	0.303						
>=35					0.74 (0.50,1.08)	0.121						
**Mother’s education**												
No education					1.84 (1.24,2.71)	0.002	1.48 (1.02,2.16)	0.037	1.18 (0.69,2.02)	0.533		
Primary					1.66 (1.13,2.43)	0.010	1.33 (0.93,1.93)	0.116	1.05 (0.62, 1.77)	0.847		
Secondary					1.38 (0.94,2.05)	0.099	1.18 (0.81,1.72)	0.377	0.81 (0.47, 1.39)	0.445		
Higher					1		1		1			
**Mother’s occupation**												
Not working					1		1		1		1	
Non agriculture					1.02 (0.93,1.12)	0.601	1.01 (0.91,1.12)	0.887	0.86 (0.74, 1.01)	0.054	0.84 (0.73,0.97)	0.022
Agriculture					1.22 (1.12,1.33)	p < 0.001	1.11 (0.99,1.23)	0.051	1.02 (0.89, 1.18)	0.734	1.02 (0.89,1.17)	0.738
**ANC Visit**												
None					1		1		1		1	
1–3					0.87 (0.79,0.94)	0.002	0.93 (0.84,1.03)	0.159	0.92 (0.81,1.04)	0.214	0.90 (0.79,1.02)	0.108
4+					0.71 (0.64,0.79)	p < 0.001	0.74 (0.66,0.82)	p < 0.001	0.78 (0.67,0.91)	0.002	0.74 (0.64,0.87)	p < 0.001
**Maternal BMI (**kg/m^2^)												
< 18.5					1		1		1		1	
18.5 to 24.9					0.96 (0.89,1.04)	0.347	0.93 (0.85,1.02)	0.116	0.64 (0.57,0.72)	p < 0.001	0.65 (0.58,0.73)	p < 0.001
25 +					0.79 (0.66,0.95)	0.017	0.79 (0.65,0.96)	0.019	0.39 (0.29,0.53)	p < 0.001	0.39 (0.28,0.52)	p < 0.001
**Husband’s education**												
No education					1.33 (1.04,1.69)	0.019	1.51 (1.17, 1.93)	0.001	1.06 (0.76, 1.47)	0.738		
Primary					1.22 (0.96,1.54)	0.103	1.38 (1.08,1.75)	0.009	0.87 (0.62,1.21)	0.411		
Secondary					0.99 (0.78,1.27)	0.977	1.14 (0.89,1.47)	0.289	1.12 (0.79, 1.57)	0.508		
Higher					1		1		1			
**Husband’s occupation**												
Not working					0.65 (0.52,0.80)	p < 0.001	0.75 (0.61,0.92)	0.007	1.06 (0.81, 1.38)	0.654	1.08 (0.83,1.40)	0.571
Agriculture					1.01 (0.92,1.12)	0.782	1.11 (0.99,1.24)	0.066	1.16 (0.98, 1.36)	0.077	1.16 (0.99, 1.35)	0.062
Non agriculture					1		1		1		1	
**Listening to radio**												
Not at all					1							
Yes					0.99 (0.91,1.07)	0.820						
**Watching television**												
Not at all					1		1		1		1	
Yes					0.84 (0.75,0.94)	0.002	0.92 (0.82,1.03)	0.152	0.78 (0.66,0.92)	0.003	0.71 (0.61,0.84)	p < 0.001
***Household factors***												
**Wealth index**												
Poor							1.33 (1.13,1.56)	0.001	1.35 (1.14,1.60)	p < 0.001	1.40 (1.18,1.66)	p < 0.001
Middle							1.15 (0.95,1.38)	0.143	1.24 (1.03,1.50)	0.022	1.27 (1.05,1.53)	0.012
Rich							1		1	1	1	
**Household Size**												
1–4							0.94 (0.84,1.04)	0.231				
>=5							1					
***Community Level Factors***												
**Residence**												
Urban									1		1	
Rural									0.57 (0.44,0.72)	p < 0.001	0.58 (0.46,0.73)	p < 0.001
**Region**												
Agrarian									0.96 (0.79,1.17)	0.711		
Pastoralist									0.95 (0.78,1.17)	0.654		
City administrations									1			
**Ecological Zone**												
< 1500									1.61 (1.30,1.99)	p < 0.001	1.61 (1.30,1.99)	p < 0.001
1500–2500									1.18 (0.96,1.46)	0.105	1.17 (0.96,1.45)	0.115
> 2500									1		1	

AOR (Adjusted Odds Ratio); COR (Crude Odds Ratio), WASH (Water, Sanitation, and Hygiene), LL: Log-likelihood

Model 0: Empty model (-LL: -11893.181), ICC(%): 3.27, AIC: 23790.36, BIC: 23807.1

Model 1: WASH variables only (-LL: -11137.13), ICC(%): 2.87, AIC: 22294.26, BIC: 22377.31

Model 2: WASH + Child related factors (from model 0 with p < 0.20) (-LL: -15302.49), ICC(%): 3.10, AIC: 30654.98, BIC: 30858.13

Model 3: WASH + Child related factors (from model 2 with p < 0.20) + Parental factors (from model 0 with p < 0.20) (-LL: -10101.304)

Model 4: WASH + Child related factors s(p < 0.20) + Parental factors (from model 0 with p < 0.20) + Household factors (-LL: -4938.8883), ICC(%): 2.08; AIC: 10019.7, BIC: 10328.02

Model 5: WASH + Child related factors (p < 0.20) + Parental factors (from model 0 with p < 0.20) + Household factors (from model 4 with p < 0.2) + Community level factors (-LL: -4940.3857), ICC(%):1.56, AIC: 9968.771, BIC: 10299.64

Model 6: (final model): The final model includes only variables with a p < 0.2 from model 5. (-LL: -4990.04 ), ICC(%): 1.70, AIC: 10050.08, BIC: 10313.57

^a^Variables such as household drinking water service and combined sanitation facility were removed by the model due to multicollinearity

# WASH factors significant associated with wasting (p < 0.05)

## Discussion

A selection of socioeconomic and demographic variables as controlling factors were significantly associated with the prevalence of stunting and wasting among children in Ethiopia as demonstrated above. Early childhood linear growth is a strong indicator of healthy growth and is linked to child development in several domains. One of the factors affecting nutritional status in childhood is poor WASH. The lack of access to WASH may also affect children’s health and well-being in various ways (for example, through repeated exposure of diarrheal infections), which potentially increases the risk of wasting. This study identified the association between WASH factors and childhood undernutrition in Ethiopia. This study’s overall prevalence of stunting and wasting was 47.29% and 10.98%, respectively.

Stunting was associated with latrine facilities, sources of drinking water, and household flooring. All WASH factors (sanitation facility, sources of drinking water, disposal of the child’s stool, and time to the water source) were individually related to stunting among Ethiopian children under the age of five. However, only a few WASH variables remained statistically significant after correcting potential confounders.

Under-fives who lived with families where open defecation was practised, were more likely to be stunted. This finding agrees with recent findings from the Ethiopian research project entitled GROW (Growing Nutrition for Mothers and Children), which found that open defecation was strongly connected with stunting in Ethiopia [40]. Open defecation, which may be associated with poor sanitation and hygiene, is a significant predictor of a wide range of population diseases, including bacaterial and viral diarrhoeal illnesses such as typhoid, cholera, hepatitis, and are among childhood infectious conditions that are associated with undernutrition [41]. A study in India showed that children from households that defecate in the open have a 14% higher likelihood of being stunted than children from households that use improved latrines [42]. Another study by Rah et al. reported that compared with open defecation, household access to a latrine facility was associated with a 16–39% reduced odds of stunting among children aged 0–23 months, after adjusting for all potential confounders [43]. This finding indicated that children living in locations where open defecation was common were more vulnerable to stunting, and this is due to repeated episodes of diarrheal illnesses in these localities. Repeated episodes of diarrhea are known to be linked with poor WASH. Diarrheal illness contributes to undernutrition by reducing food intake, nutrient absorption, and increasing the catabolism of nutrient stores.

Previous literature has widely acknowledged the relationship between unimproved sanitation and stunting [42, 44, 45]. This finding was reaffirmed in the current study as children from households having unimproved latrines were more likely to be stunted. Analysis of a variety of cross-sectional studies based on data from the Demographic and Health Surveys (DHS) from other countries have also indicated that improved sanitation is important for the linear growth of children. For instance, data from 172 Demographic and Health Surveys (DHS) showed that the odds of being stunted were lower in households with access to improved sanitary facilities [46]. On the other hand, studies from India and Indonesia reported that open defecation was negatively associated with height-for-age measures [47, 48].

It was hypothesised that improved water sources could have been linked to moderate-to-severe stunting [49, 50]. In this study however, we observed a negative association between unimproved sources of drinking water and lower odds of being stunted. In the bivariable multilevel model children from households that use unimproved sources of drinking water had higher odds of being stunted (OR: 1.39, 95% CI: 1.32–1.46, *p-value < 0.001*). This association did not remain consistent after adjustingg for individual and community variables. In line with this finding, Torlesse et al. reported that stunting was not associated with the household’s source of drinking water [48]. Another study from Indonesia also reported that the source of drinking water was not predictive of stunting [51]. On the other hand, the unavailability of water was reported as a significant factor associated with moderate and severe stunting [44, 52]. A cross-sectional study from Kersa, Ethiopia reported that households using non-piped water and that did not treat drinking water were at higher odds of being stunted [AOR: 1.5, 95% CI (1.07–2.00)], and [AOR: 1.9, 95% CI (1.31–2.85)], respectively [8]. The difference in the definitions of the terms across studies the and quality of drinking water including microbiological features, might have contributed to this inconsistent association. Furthermore, large experimental studies, such as the WASH-Benefits Bangladesh [53], the WASH-Benefits Kenya [21] and the Sanitation Hygiene Infant Nutrition Efficacy (SHINE) trials in Zimbabwe [22] found no effects of any WASH intervention on child linear growth. The evidence for the association between the water source and stunting remains unclear [48]. A study by Cumming and Cairncross suggested that the causal pathways linking poor WASH to child developmental are complex, spanning multiple routes [44].

The causes of stunting are multifactorial and interlinked, as depicted by various conceptual frameworks [54]. Our findings support the idea that nutrition interventions alone are unlikely to improve childhood nutritional problems. Importantly, in this study, children from households with dirt floors had higher odds of being stunted than their counterparts. Cross-sectional data from 54 Demographic and Health Surveys showed that improved housing was associated with 12% lower odds of stunting [55]. Current evidence clearly shows that the housing environment has significantly impacts children’s growth and development [55, 56].

WASH factors such as the type of latrine facilities, the disposal of the child’s stool, and household flooring were significantly associated with wasting in the unadjusted bivariable multilevel model. Still, they were all diminished after adjusting for all potential confounders. In this study, the type of latrine facilities, the disposal of the child’s stool, and household flooring were not significantly associated with childhood wasting in the final model. One possible explanation for the lack of association in our analysis is that there may be a weak link between safe and adequate coverage of improved water different parts of the country. Similar findings were reported by studies conducted in multiple countries including Ethiopia [10], Guatemala [57], and Nepal [58]. For instance, a study conducted in Ethiopia showed that access to improved individual water, sanitation, handwashing and combined WASH facilities were not predictive of wasting in children when adjusted for confounders [10]. Conversely, a study conducted among Afghan children found increased odds of wasting among children with unimproved water sources to children withen who had access to protected water sources [59].

We also observed an inversely significant relationship between the use of unimproved sources of drinking water and a lower likelihood of wasting. The lack of relevant data on bacteriological quality, chemical, physical properties of drinking water, and the duration of households with improved water sources in the EDHS datasets may explain the inverse association we found between the use of unimproved sources of drinking water and a lower risk of wasting. In this case, our definition did not meet the criteria for a safely managed drinking water service, which means that households must use an improved source that is: accessible on premises, available when needed, and free of contamination, as stated by the new global indicators for drinking water definition of WHO/UNICEF Joint Monitoring Programme for Water Supply and Sanitation [60]. Furthermore in Ethiopia, most household residents in both urban (88%) and rural (92%) areas report that they do not treat their water prior to drinking [29].

The current study used representative population-based data with a high response rate and was analyzed by adjusting for weighting, clustering, and stratification. There are some limitations to this study. Firstly, cause-effect relationships could not be established in the current study due to the study design. Secondly, due to a lack of relevant data in EDHS, the effects of the microbiological, chemical, and physical properties of drinking water sources on child growth outcomes is unknown. Thirdly, because the only access to WASH facilities was considered, the true association between WASH practices and child growth outcomes may be underestimated. Fourthly, as is often the situation in observational cross-sectional studies, it is difficult to rule out biases, included the recall biases in the current study. Fifthly, our study may have been hampered by unmeasured confounders, such as dietary practices and food security problems.

### Conclusion

The present study showed that children from households that defecated in the open and had unimproved latrine facilities were more likely to be stunted. Similarly, when adjusted, a significant association of children’s linear growth failure (stunting) among under-five children was found among children living in households with dirt floors. On the other hand, there was no evidence of a relationship between wasting and the type of latrine facilities, the disposal of the child’s stool, or household flooring. Having unimproved drinking water sources was associated with lower odds of being wasted. Policies and interventions should target improved sanitation and housing conditions in order to prevent child stunting. Finally and to enlighten more on these issues in Ethiopia, further longitudinal and interventional studies are warranted, to examine the effects of access to WASH facilities and/or household WASH practices and their association with child nutritional status and development.

## Electronic supplementary material

Below is the link to the electronic supplementary material.

Supplementary Material 1

Supplementary Material 2

## Declarations

### Data availability

The dataset was demanded and retrieved from the DHS website https://dhsprogram.com after formal online registration and submission of the project title and detailed description.

## Abbreviations

AOR: Adjusted odds ratio
ANC: antenatal care visits
BMI: body mass index
CI: Confidence interval
EDHS: Ethiopian Demographic and Health Surveys
SNNP: Southern Nations and Nationalities and People
WASH: Water, sanitation, and hygiene
WHO: World Health Organization
