# Supplementary material for: Association between water, sanitation and hygiene (WASH) and child undernutrition in Ethiopia: a hierarchical approach
Source: BMC Public Health. 2022 Oct 19;22:1943. doi: 10.1186/s12889-022-14309-z (PMC9583486; doi:10.1186/s12889-022-14309-z)
Supplement: Supplementary file 1 — Supplementary Material 1 [file 12889_2022_14309_MOESM1_ESM.docx]

**Additional File 1: Multilevel bivarible binary logistic regression analysis on the effects of WASH and other factors on prevalence of stunting among under-5 children, Ethiopia (n=33,744), 2000–2016.**

| **Characteristics** | **Stunting** | | **Crude OR, 95%CI** | **p-value** |
| --- | --- | --- | --- | --- |
|  | **Yes** | **No** |  |  |
| ***WASH factors*** |  |  |  |  |
| **Latrine facility (n= 33,154)** |  |  |  |  |
| Improved | 1,453 | 2,341 | 1 |  |
| Unimproved | 4,358 | 6,217 | 1.61 (1.49,1.74) | p<0.001 |
| Open defecation | 9,864 | 8,918 | 2.13 (1.98,2.29) | p<0.001 |
| **Source of drinking water (n=33,147)** |  |  |  |  |
| Improved | 5,575 | 7,215 | 1 |  |
| Unimproved | 10,098 | 10,258 | 1.39 (1.32,1.46) | p<0.001 |
| **Child stool disposal (n=33,637)** |  |  |  |  |
| Safe | 2,908 | 4,183 | 1 |  |
| Unsafe | 12,999 | 13,546 | 1.55 (1.46,1.64) | p<0.001 |
| **Household flooring** |  |  |  |  |
| Improved | 759 | 1,957 | 1 |  |
| Unimproved | 95 | 15,821 | 2.82 (2.60,3.05) | p<0.001 |
| **Time to get a water source** |  |  |  |  |
| On-premise | 465 | 1,304 | 1 |  |
| ≤ 30 min | 9,540 | 10,005 | 2.80 (2.53,3.09) | p<0.001 |
| 31-60 min | 3,346 | 3,548 | 2.82 (2.53, 3.14) | p<0.001 |
| >60 min | 2,521 | 2,806 | 2.76 (2.47, 3.08) | p<0.001 |
| **Household drinking water service** |  |  |  |  |
| Basic drinking water service | 3,688 | 4,860 | 1 |  |
| Limited drinking water service | 1,8102 | 2,233 | 1.25 (1.15,1.36) | p<0.001 |
| Poor drinking water service | 10,466 | 10,692 | 1.42 (1.34,1.49) | p<0.001 |
| **Combined sanitation facility** |  |  |  |  |
| Improved W + Improved S | 801 | 1,540 | 1 |  |
| Either one improved | 5,424 | 6,476 | 2.19 (2.01,2.39) | p<0.001 |
| Unimproved W + Unimproved S | 9,447 | 9,457 | 2.57 (2.36,2.79) | p<0.001 |
| ***Child Factors*** |  |  |  |  |
| ***Childhood infections*** |  |  |  |  |
| **Diarrhea (n=33,697)** |  |  |  |  |
| Yes | 2,978 | 2,780 | 1.25 (1.17,1.33) | p<0.001 |
| No | 12,978 | 14,973 | 1 |  |
| **Fever** |  |  |  |  |
| Yes | 3,409 | 3,444 | 1.19 (1.13,1.26) | p<0.001 |
| No | 12,526 | 14,315 | 1 |  |
| **ARI (n=33,744)** |  |  |  |  |
| Yes | 575 | 779 | 0.92 (0.82,1.04) | 0.206 |
| No | 15,382 | 17,006 | 1 |  |
| **Sex (n=33,744)** |  |  |  |  |
| Male | 8,456 | 8,715 | 1 |  |
| Female | 7,501 | 9,070 | 0.89 (0.84,0.93) | p<0.001 |
| **Age (months)** |  |  |  |  |
| < 12 | 1,455 | 5,546 | 1 |  |
| 12-23 | 3,224 | 3,339 | 4.19 (3.85,4.55) | p<0.001 |
| ≥24 to 59 | 11,278 | 8,901 | 5.43 (5.05,5.83) | p<0.001 |
| **Birth order** |  |  |  |  |
| Firstborn | 2,682 | 3,324 | 1 |  |
| 2-4 | 6,772 | 7,775 | 1.13 (1.07,1.21) | p<0.001 |
| 5 or higher | 6,504 | 6,687 | 1.34 (1.25,1.43) | p<0.001 |
| **Birth interval** |  |  |  |  |
| < 33 months | 10,789 | 12,487 | 1 |  |
| ≥33 months | 5,168 | 5,299 | 1.15 (1.10,1.22) | p<0.001 |
| **Size of child at birth** |  |  |  |  |
| Larger | 4,533 | 5,898 | 1 |  |
| Average | 6,064 | 7,171 | 1.11 (1.05,1.17) | p<0.001 |
| Small | 5,324 | 4,654 | 1.46 (1.37,1.55) | p<0.001 |
| **Currently breastfeeding** |  |  |  |  |
| Yes | 11,672 | 13,359 | 1 |  |
| No | 4,286 | 4,426 | 1.17 (1.11,1.23) | p<0.001 |
| **Early initiation of breastfeeding (n=25, 991)** |  |  |  |  |
| Yes | 6,359 | 7,784 | 1 |  |
| No | 5,871 | 5,977 | 1.14 (1.08,1.20) | p<0.001 |
| **Received measles (n=29,269)** |  |  |  |  |
| Yes | 5,491 | 10,090 | 1 |  |
| No | 8,411 | 5,276 | 0.81 (0.77,0.85) | p<0.001 |
| **Basic vaccine (n=28,859)** |  |  |  |  |
| Yes | 2,776 | 2,831 | 1 |  |
| No | 10,910 | 12,339 | 0.94 (0.88,0.99) | 0.033 |
| ***Parental factors*** |  |  |  |  |
| **Mother’s age** |  |  |  |  |
| <18 | 119 | 159 | 1 |  |
| 18-24 | 3,518 | 4,299 | 1.37 (1.04,1.79) | 0.023 |
| 25-34 | 8,113 | 9,009 | 1.55 (1.18,2.03) | 0.001 |
| ≥35 | 4,206 | 4,318 | 1.72 (1.31,2.26) | p<0.001 |
| **Mother’s education** |  |  |  |  |
| No education | 12,392 | 12,204 | 5.56 (4.36,7.09) | p<0.001 |
| Primary | 3,110 | 4,287 | 3.79 (2.96, 4.85) | p<0.001 |
| Secondary | 381 | 971 | 1.85 (1.42, 2.41) | p<0.001 |
| Higher | 73 | 323 | 1 |  |
| **Mother’s occupation (n=33,639)** |  |  |  |  |
| Not working | 7,327 | 9,073 | 1 |  |
| Non agriculture | 3,147 | 3,926 | 0.95 (0.89,1.01) | 0.110 |
| Agriculture | 5,427 | 4,736 | 1.54 (1.46,1.63) | p<0.001 |
| **ANC Visit (n=23,125)** |  |  |  |  |
| None | 6,598 | 6,616 | 1 |  |
| 1-3 | 2,221 | 3,102 | 0.71 (0.66,0.76) | p<0.001 |
| 4+ | 1,581 | 3,005 | 0.48 (0.44,0.52) | p<0.001 |
| **Maternal BMI (**kg/m^2^) (n=33,547) |  |  |  |  |
| <18.5 | 3,561 | 3,492 | 1 |  |
| 18.5 to 24.9 | 11,868 | 13,213 | 0.89 (0.84,0.94) | p<0.001 |
| 25 + | 439 | 972 | 0.37 (0.32,0.41) | p<0.001 |
| **Husband’s education (n=32,879)** |  |  |  |  |
| No education | 9,269 | 8,564 | 3.65 (3.17,4.19) | p<0.001 |
| Primary | 5,138 | 6,480 | 2.75 (2.38,3.17) | p<0.001 |
| Secondary | 946 | 1,594 | 1.69 (1.44,1.97) | p<0.001 |
| Higher | 211 | 673 | 1 |  |
| **Listening to radio** |  |  |  |  |
| Not at all | 11,009 | 11,148 | 1 |  |
| Yes | 4,939 | 6,638 | 0.75 (0.72,0.79) | p<0.001 |
| **Watching television** |  |  |  |  |
| Not at all | 13,694 | 14,125 | 1 |  |
| Yes | 2,251 | 3,649 | 0.52 (0.49,0.55) | p<0.001 |
| ***Household factors*** |  |  |  |  |
| **Wealth index** |  |  |  |  |
| Poor | 5,151 | 5,696 | 1.71 (1.59,1.81) | p<0.001 |
| Middle | 2,187 | 2,811 | 1.52 (1.39,1.65) | p<0.001 |
| Rich | 2,983 | 5,103 | 1 |  |
| **Household Size** |  |  |  |  |
| 1-4 | 3,648 | 4,449 | 0.86 (0.82,0.91) | p<0.001 |
| ≥ 5 | 12,309 | 13,337 | 1 |  |
| ***Community Level Factors*** |  |  |  |  |
| **Residence** |  |  |  |  |
| Urban | 1,262 | 2,377 | 1 |  |
| Rural | 14,695 | 15,409 | 2.29 (2.14,2.46) | p<0.001 |
| **Region** |  |  |  |  |
| Agrarian | 9,357 | 9,007 | 1.91 (1.77,2.05) | p<0.001 |
| Pastoralist | 6,396 | 8,194 | 1.78 (1.63,1.93) | p<0.001 |
| City | 203 | 584 | 1 |  |
| **Ecological Zone** |  |  |  |  |
| <1500 | 1,376 | 2,225 | 0.65 (0.58,0.73) | p<0.001 |
| 1500-2500 | 7,244 | 9,588 | 0.73 (0.66,0.82) | p<0.001 |
| >2500 | 1,700 | 1,797 | 1 |  |
