# Supplementary material for: Association between water, sanitation and hygiene (WASH) and child undernutrition in Ethiopia: a hierarchical approach
Source: BMC Public Health. 2022 Oct 19;22:1943. doi: 10.1186/s12889-022-14309-z (PMC9583486; doi:10.1186/s12889-022-14309-z)
Supplement: Supplementary file 2 — Supplementary Material 2 [file 12889_2022_14309_MOESM2_ESM.docx]

**Additional File 2: Multilevel bivarible binary logistic regression analysis on the effects of WASH and other factors on prevalence of wasting among under-5 children, Ethiopia (n=33,763), 2000–2016.**

| **Characteristics** | **Wasting** | | **Crude OR, 95%CI** | **p-value** |
| --- | --- | --- | --- | --- |
|  | **Yes** | **No** |  |  |
| ***WASH factors*** |  |  |  |  |
| **Latrine facility** |  |  |  |  |
| Improved | 318 | 3,476 | 1 |  |
| Unimproved | 928 | 9,635 | 1.11 (0.98,1.26) | 0.087 |
| Open defecation | 2,395 | 16,418 | 1.78 (1.60,1.98) | p<0.001 |
| **Source of drinking water (n=33,166)** |  |  |  |  |
| Improved | 1,367 | 11,453 | 1 |  |
| Unimproved | 2,275 | 18,069 | 1.17 (1.09,1.25) | p<0.001 |
| **Child stool disposal (n=33,656)** |  |  |  |  |
| Safe | 576 | 6,519 | 1 |  |
| Unsafe | 3,107 | 23,453 | 1.50 (1.37,1.65) | p<0.001 |
| **Household flooring** |  |  |  |  |
| Improved | 198 | 2,511 | 1 |  |
| Unimproved | 3,508 | 27,537 | 1.87 (1.65,2.11) | p<0.001 |
| **Time to get a water source** |  |  |  |  |
| On-premise | 140 | 1,626 | 1 |  |
| ≤ 30 min | 2,134 | 17,415 | 1.59 (1.37, 1.86) | p<0.001 |
| 31-60 min | 786 | 6,124 | 1.78 (1.51,2.09) | p<0.001 |
| >60 min | 636 | 4,695 | 2.06 (1.74,2.43) | p<0.001 |
| **Household drinking water service** |  |  |  |  |
| Basic drinking water service | 906 | 7,656 | 1 |  |
| Limited drinking water service | 447 | 3,605 | 1.26 (1.12,1.43) | p<0.001 |
| Poor drinking water service | 2,355 | 18,791 | 1.22 (1.12,1.33) | p<0.001 |
| **Combined sanitation facility** |  |  |  |  |
| Improved W + Improved S | 166 | 2,180 | 1 |  |
| Either one improved | 1,353 | 10,567 | 1.82 (1.58,2.09) | p<0.001 |
| Unimproved W + Unimproved S | 2,123 | 16,774 | 1.88 (1.64,2.15) | p<0.001 |
| ***Child factors*** |  |  |  |  |
| ***Childhood infections*** |  |  |  |  |
| **Diarrhea (n=33,716)** |  |  |  |  |
| Yes | 916 | 4,850 | 1.64 (1.51,1.79) | p<0.001 |
| No | 2,784 | 25,166 | 1 |  |
| **Fever** |  |  |  |  |
| Yes | 1,042 | 5,820 | 1.56 (1.44,1.69) | p<0.001 |
| No | 2,662 | 24,189 |  |  |
| **ARI** |  |  |  |  |
| Yes | 177 | 1,186 | 1.29 (1.09,1.53) | 0.002 |
| No | 3,532 | 28,866 | 1 |  |
| **Sex** |  |  |  |  |
| Male | 2,063 | 15,143 | 1 |  |
| Female | 1,646 | 14,910 | 0.82 (0.76,0.87) | p<0.001 |
| **Age (months)** |  |  |  |  |
| < 12 | 1,116 | 5,858 | 1 |  |
| 12-23 | 1,009 | 5,565 | 0.89 (0.81,0.98) | 0.019 |
| ≥24 to 59 | 1,584 | 18,629 | 0.45 (0.42,0.49) | p<0.001 |
| **Birth order** |  |  |  |  |
| Firstborn | 575 | 5,428 | 1 |  |
| 2-4 | 1,558 | 12,995 | 1.16 (1.05,1.28) | 0.003 |
| 5 or higher | 1,576 | 11,629 | 1.35 (1.22,1.49) | p<0.001 |
| **Birth interval** |  |  |  |  |
| < 33 months | 2,508 | 20,767 | 1 |  |
| ≥33 months | 1,200 | 9,286 | 1.01 (0.94,1.09) | 0.695 |
| **Size of child at birth** |  |  |  |  |
| Larger | 924 | 9,529 | 1 |  |
| Average | 1,364 | 11,876 | 1.19 (1.09,1.30) | p<0.001 |
| Small | 1,414 | 8,555 | 1.79 (1.63,1.95) | p<0.001 |
| **Currently breastfeeding** |  |  |  |  |
| Yes | 22,012 | 3,029 | 1 |  |
| No | 8,040 | 680 | 0.69 (0.63,0.74) | p<0.001 |
| **Early** **initiation of breastfeeding** |  |  |  |  |
| Yes | 1,620 | 12,515 | 1 |  |
| No | 1,486 | 10,355 | 1.04 (0.96,1.12) | 0.314 |
| **Received measles** |  |  |  |  |
| Yes | 993 | 9,779 | 1 |  |
| No | 2,378 | 16,105 | 1.61 (1.49,1.74) | p<0.001 |
| **Basic vaccine** |  |  |  |  |
| Yes | 502 | 5,101 | 1 |  |
| No | 2,819 | 20,424 | 1.61 (1.46,1.78) | p<0.001 |
| ***Parental factors*** |  |  |  |  |
| **Mother's age** |  |  |  |  |
| <18 | 37 | 238 | 1 |  |
| 18-24 | 885 | 6,934 | 0.71 (0.51,1.01) | 0.051 |
| 25-34 | 1,796 | 15,329 | 0.64 (0.46,090) | 0.011 |
| ≥35 | 990 | 7,552 | 0.69 (0.49,0.98) | 0.038 |
| **Mother's education** |  |  |  |  |
| No education | 2,940 | 21,682 | 2.70 (1.87,3.90) | p<0.001 |
| Primary | 658 | 6,741 | 1.91 (1.31, 2.78) | 0.001 |
| Secondary | 85 | 1,260 | 1.24 (0.82, 1.87) | 0.306 |
| Higher | 25 | 369 | 1 |  |
| **Mother's occupation** |  |  |  |  |
| Not working | 1,795 | 14,618 | 1 |  |
| Non agriculture | 668 | 6,417 | 0.74 (0.67,0.81) | p<0.001 |
| Agriculture | 1,238 | 8,920 | 1.02 (0.94, 1.11) | 0.541 |
| **ANC Visit** |  |  |  |  |
| None | 1,799 | 11,403 | 1 |  |
| 1-3 | 639 | 4,673 | 0.84 (0.77,0.93) | p<0.001 |
| 4+ | 444 | 4,146 | 0.52 (0.47,0.58) | p<0.001 |
| **Maternal BMI (**kg/m^2^) |  |  |  |  |
| <18.5 | 1,118 | 5,924 | 1 |  |
| 18.5 to 24.9 | 2,481 | 22,630 | 0.55 (0.51,0.59) | p<0.001 |
| 25 + | 77 | 1,335 | 0.26 (0.21,0.32) | p<0.001 |
| **Husband's education** |  |  |  |  |
| No education | 2,261 | 15,615 | 1.82 (1.49,2.23) | p<0.001 |
| Primary | 1,092 | 10,501 | 1.24 (1.01,1.54) | 0.037 |
| Secondary | 193 | 2,347 | 1.18 (0.94,1.49) | 0.148 |
| Higher | 62 | 822 | 1 |  |
| **Listening to radio** |  |  |  |  |
| Not at all | 1,044 | 19,510 | 1 |  |
| Yes | 2,661 | 10,537 | 0.66 (0.61,0.71) | p<0.001 |
| **Watching television** |  |  |  |  |
| Not at all | 3,257 | 24,583 | 1 |  |
| Yes | 451 | 5,448 | 0.53 (0.47,0.58) | p<0.001 |
| ***Household factors*** |  |  |  |  |
| **Wealth index** |  |  |  |  |
| Poor | 1,383 | 9,497 | 1.95 (1.76,2.16) | p<0.001 |
| Middle | 518 | 4,480 | 1.52 (1.33,1.74) | p<0.001 |
| Rich | 582 | 7,490 | 1 |  |
| **Household Size** |  |  |  |  |
| 1-4 | 866 | 7,228 | 0.89 (0.82,0.97) | 0.006 |
| ≥ 5 | 2,843 | 22,824 | 1 |  |
| ***Community-Level Factors*** |  |  |  |  |
| **Residence** |  |  |  |  |
| Urban | 270 | 3,368 | 1 |  |
| Rural | 3,439 | 26,684 | 1.61 (1.45,1.80) | p<0.001 |
| **Region** |  |  |  |  |
| Agrarian | 2,107 | 16,257 | 1.55 (1.38,1.74) | p<0.001 |
| Pastoralist | 1,555 | 13,055 | 1.55 (1.37,1.76) | p<0.001 |
| City | 47 | 740 | 1 |  |
| **Ecological Zone** |  |  |  |  |
| <1500 | 472 | 3,151 | 2.01 (1.68,2.41) | p<0.001 |
| 1500-2500 | 1,699 | 15,130 | 1.23 (1.03,1.48) | p<0.001 |
| >2500 | 312 | 2,186 | 1 |  |
